# Reducing stigma and increasing competence working with mental illness: Adaptation of a contact-based program for osteopathic medical students to a virtual, active learning format

**DOI:** 10.1080/10872981.2022.2151069

**Published:** 2022-11-24

**Authors:** Julia R. Van Liew, Chunfa Jie, Jeritt R. Tucker, Lisa Streyffeler

**Affiliations:** a Department of Behavioral Medicine, Medical Humanities, and Bioethics, Des Moines University, Des Moines, IA, USA; b Department of Biochemistry and Nutrition, Des Moines University, Des Moines, IA, USA

**Keywords:** Mental illness, stigma reduction, psychiatry education, contact-based education, virtual learning

## Abstract

**Purpose:**

Contact-based education, offering meaningful contact with individuals living in recovery with mental illness, reduces stigma. This study evaluated the effectiveness of the National Alliance on Mental Illness Provider Education Program (NAMI PEP) when implemented as a curricular requirement across two cohorts of third-year osteopathic medical students, comparing traditional, passive learning and active, online delivery formats.

**Materials and Methods:**

Participants were two cohorts of third-year medical students (Cohort 1 n = 186; Cohort 2 n = 139; overall N = 325) who completed questionnaires measuring affect, beliefs, and behaviors toward patients with mental illness at pre-program, 1-week follow-up, and 6-month follow-up. For Cohort 1, the existing community-based NAMI PEP was implemented. For Cohort 2, the program was adapted to an online, active learning format tailored to medical students, and an additional 3-month follow-up assessment was added to better identify intermediate-term effects.

**Results:**

The NAMI PEP was associated with longitudinal improvements in target outcomes, with enhanced effects with the adapted curriculum in Cohort 2. At 6-month follow-up, students reported less stigma, fewer stereotyping negative attitudes, and lower anxiety treating patients with mental illness. They also reported increased confidence integrating psychiatry into routine care and increased competence in principles of collaborative mental health treatment.

**Conclusions:**

This study demonstrates the longitudinal effectiveness of the NAMI PEP across two cohorts of medical students, with strengthened effects observed when the program is tailored to contemporary medical education.

Consistent with mental health stigma prevalent in the general public, physicians report negative affect (emotions), unfavorable attitudes, and low perceived self-efficacy related to working with patients with mental illness (MI) [1–4]. Physician mental health stigma is associated with fewer specialty referrals, fewer medication prescriptions, and lower expectations of adherence for patients with psychiatric diagnoses [5]. Undergraduate medical school education represents a meaningful opportunity for mental health stigma reduction, with potential impact on future physicians across the workforce [4]. Unfortunately, traditional medical school education appears to have limited effect on – and may even exacerbate – negative attitudes, emotional reactions, and behavioral intentions toward patients with MI [4,6–9]. This is thought to stem from recognized constraints of typical psychiatry education including limited integration of psychiatry with other clinical medical disciplines, clerkship training in highly select settings (e.g., inpatient psychiatric units only), and exposure to isolated phases of the illness trajectory (e.g., acute psychiatric crises only) [4].

Contact-based educational programs, consisting of focused social interactions with individuals with MI, represent promising adjunctive training opportunities for changing negative affect, attitudes, and behavioral intentions related to working with patients with MI [10]. Optimal contact-based learning offers multiple opportunities for high-quality, meaningful social contact that occurs outside of routine patient care [11,12]. Additional key elements of effective contact-based learning include facilitators with first-person experiences, contradiction of stereotypes, and teaching communication and behavioral skills for future patient interactions [11]. In health professional students, contact with more senior providers sharing personal experiences of MI may be particularly salient for stigma reduction [13–15].

Within and beyond the field of psychiatry, existing research evaluating direct contact has several noted limitations [16]. Issues include largely unstandardized interventions, sparse assessment of longitudinal impact, difficulties with scalability, and emphasis on changes in beliefs and attitudes instead of behavioral outcomes crucial to future patient care [12,16–18]. These limitations hinder the implementation and dissemination potential of such programming. Of particular importance to implementation needs in medical schools, existing contact-based psychiatry education offers limited use of teaching and learning methods characteristic of contemporary academic medicine, such as flipped classroom, active learning, and virtual delivery strategies [19]. The increased need for virtual learning in medical schools has been further accelerated by the COVID-19 pandemic. At the same time, the degree to which the benefits of in-person contact-based mental health education extends to contact facilitated through virtual platforms remains unclear. Changing the nature of the interpersonal context from a shared physical space to a shared virtual space may alter its impact in meaningful ways that could facilitate or inhibit influential factors such as engagement and psychological safety [20,21]. There is initial evidence that stigma reduction benefits are maintained when changing an in-person contact-based program to a virtual format [13,14]. However, studies that explicitly compare the exact same set of multidimensional outcome measures across in-person and virtual formats and include longitudinal analyses of effects are lacking [18]. The National Alliance on Mental Illness Provider Education Program (NAMI PEP) offers one validated, contact-based educational intervention that may be useful for such a comparison.

## NAMI PEP

The NAMI PEP is a standardized, 15-hour contact-based program aiming to increase health-care professionals’ delivery of patient-centered and collaborative care for patients with MI. It entails small-group instruction by a team of three NAMI-trained facilitators representing a person living well in mental health recovery, a family member of a person with MI, and a health-care provider with personal or familial experience with MI. It promotes greater understanding of the impact of MI on individuals and their families, with facilitators sharing personal, familial, and clinical experiences with MI and recovery. It also includes didactic presentations, small-group discussions, clinical scenarios, and experiential exercises.

Although originally designed for practicing health-care professionals, the NAMI PEP is a promising program for medical students. It incorporates each of the above-referenced optimal conditions for contact-based education and offers inherent implementation and scale potential as a standardized program with existing delivery infrastructure through NAMI affiliates in communities across the USA. In a non-randomized, controlled trial in a self-selected medical student sample, the NAMI PEP was associated with improved affect, beliefs, and behaviors towards patients with MI at one-week and 3-month follow-up [22]. While this study demonstrated initial efficacy for implementing the NAMI PEP within medical students, its brief follow-up period, elective student sample, and primary use of traditional passive learning approaches (e.g., instructors reading material aloud to students) precluded broader implications for integration and dissemination in contemporary medical education.

### The present study

The present study evaluated the longitudinal effectiveness of the NAMI PEP across two cohorts of third-year medical students. In both cohorts, the NAMI PEP was a required course, with participation in this research study of course outcomes being optional. In the first cohort (2019), we implemented the standard NAMI PEP. In the second cohort (2020), a team of medical educators, national NAMI staff, and NAMI PEP facilitators tailored the delivery of the NAMI PEP to medical students’ specific educational needs and to reflect current pedagogical approaches in academic medicine. Specifically, we updated instructional methods to increase student engagement in learning and to integrate content with other educational experiences. Alterations included a flipped classroom model where didactic content was presented in advance via asynchronous e-modules and class time was used for active application of the material; increased amounts of pair share and breakout group discussions; increased use of case examples; integration of content with clerkship experiences; and embedded opportunities for students to document specific lessons to apply in future patient care. Additionally, we recruited psychiatry residents to serve in the health-care provider facilitator roles to enhance students’ perceived credibility and practicability of the material and provide physician modeling. Finally, we delivered the program virtually through synchronous videoconferencing (i.e., Zoom) instead of in person, utilizing recommended best practices for engaging students in this platform [20].

Overall, the third-year student samples and the content of the NAMI PEP curriculum were consistent across both cohorts, with Cohort 2 receiving the adapted delivery methods described above. Thus, the present study aimed to: 1) establish baseline pragmatic longitudinal effectiveness of the standard NAMI PEP when delivered as a required course to an entire cohort of medical students (Cohort 1; ‘standard’ curriculum), and 2) evaluate the effects of adapting instructional methods to align the program with contemporary medical school education (Cohort 2; ‘adapted’ curriculum). We hypothesized that the adapted curriculum would demonstrate enhanced outcomes across all measures. Our aims address identified gaps in the contact-based education literature, including an extended 6-month follow-up period and utilization of an existing community-based program with potential for national dissemination [16].

## Materials and methods

### Participants

During both Cohort 1 (standard) and Cohort 2 (adapted) programs, all third-year students at our osteopathic college of medicine (i.e., doctor of osteopathy degree program) were required to attend the NAMI PEP. In both cohorts, this required course occurred at the end of the third year, following pre-clinical education in psychiatry and all core clerkships including a four-week psychiatry clerkship. The course was taught simultaneously to groups of 32–37 students, each led by a separate team of NAMI facilitators, to allow for enhanced discussion. We randomly assigned students to these group sections and further randomly subdivided students into smaller breakout discussion groups for certain activities.

### Procedures

The NAMI PEP was a curricular requirement at the institution; however, participation in research evaluating program outcomes was optional. Directly preceding the start of the NAMI PEP in each cohort, we invited all enrolled students to participate in the optional research and provided time to complete a pre-program survey before beginning the course. In Cohort 1 (standard), we made an in-person verbal research invitation to students. In Cohort 2 (adapted), the flipped classroom approach necessitated an updated recruitment format, as initial exposure to the NAMI PEP now came during required pre-course asynchronous modules. To ensure completion of pre-program surveys prior to exposure to course content in these modules, students viewed a recorded research invitation prior to accessing the modules. If students agreed to participate in the study, we only provided access to the pre-program survey prior to accessing these modules. In Cohort 1, we assessed participants at pre-program, 1-week follow-up, and 6-month follow-up. Cohort 2 included these same assessment time points, with the addition of a 3-month follow-up assessment to better identify intermediate-term effects. The present study focused on changes from pre-program to 6-month follow-up. We compensated participants $5 for completion of the initial survey and $10 for each subsequent survey. All study procedures were approved by the Des Moines University IRB (IRB-2019-30). All research participation was confidential and voluntary. Names of research participants were not disclosed to course evaluators or linked to responses on surveys.

### Measures

Surveys assessed target domains of affect, beliefs, and behavioral intentions regarding mental illness. Measures were selected based on validity, brevity, and applicability to these constructs of interest. We utilized the following validated measures at all study time points across both cohorts; internal reliability (Cronbach’s α) was calculated for each scale across time points in each cohort. In the domain of *affect*, we used Day’s Mental Illness Stigma Scale – Anxiety subscale (DMISS) [23], a 7-item scale assessing anxiety concerning interacting with someone with a mental illness (Cronbach’s α Cohort 1 = 0.90, Cohort 2 = 0.87). In the domain of *beliefs*, we used: 1) the Opening Minds Scale for Health Care Providers (OMS-HC) [24,25], a 7-item subscale measuring attitudes of health-care providers toward persons with mental illness (Cronbach’s α Cohort 1 = 0.75, Cohort 2 = 0.77); and 2) the Characteristic Scale [26], a 9-item semantic differential measure of stereotyping attitudes regarding a vignette of a patient with schizophrenia (Cronbach’s α Cohort 1 = 0.86, Cohort 2 = 0.86) [27]. In the domain of *behavioral intentions*, we used: 1) the Attitudes and Confidence in the Integration of Psychiatry Scale (ACIP) [28], a 6-item subscale assessing confidence in integrating psychiatry into routine care (Cronbach’s α Cohort 1 = 0.84, Cohort 2 = 0.84); 2) Doctors’ Attitudes Toward Collaborative Care (DACC-Management) [29], a 4-item scale assessing intentions for managing mental health problems as part of routine medical care (Cronbach’s α not calculated due to dichotomous format); and 3) the Competent Caring Questionnaire (CCQ), a 14-item vignette-based measure of behavior when treating a patient with schizophrenia presenting in a general medical setting (Cronbach’s α Cohort 1 = 0.64, Cohort 2 = 0.70) [22]. Internal reliability was adequate across scales in both cohorts and in the acceptable to good range.

### Data analysis plan

We completed analyses using R software, version 4.1.1. We treated all but the time factor and categorical demographic covariates as measurements on continuous scales. We performed summary descriptive statistical calculations and simple univariate analyses (e.g., t-tests) on the outcome measures and demographic covariates. Changes in outcome measures over time were of primary interest, with the pre-program time point used as a baseline reference point for improvements. The data bear the characteristics of repeated measurements on the same participants, since more than one measurement was taken on each participant over time. Thus, it is plausible to assume that measurements on the same individual participants are correlated. Ignoring the covariance between such measurements may result in erroneous statistical inference and avoiding it by data transformation or related techniques may result in inefficient statistical inference.

The statistical technique of linear mixed modeling for repeated measures allows the covariance structure to be integrated into the modeling while accounting for the fixed time effects of the NAMI PEP as well as the randomness of participants. We built a linear mixed-effect model for repeated measures to model the time effects of the NAMI PEP while controlling for the additive effects of an optimal set of demographic and personality covariates, which were determined based on the Akaike information criterion (AIC) by searching all modeling options of the covariates. Upon determining the model’s predictor variables, we selected and evaluated several candidate covariance structures according to the study design (i.e., unequal spacing of the time points, same time points across participants, within-subject correlation over time, and convergence of model fitting), out of which we used the AIC criteria to select one final covariance structure. From the chosen final model, we assessed the impact of the NAMI PEP on the respective measures across time while accounting for covariates. We used the single-step method of the R software package, *multcomp*, to compute the adjusted p-values for the multiple comparisons of means between the baseline and later time points. The adjusted p-value cutoff of <0.05 was used to determine significance for all statistical tests. We calculated effect size (ES) using Cohen’s *d*, defining effect sizes ≥0.20 as small effects, effect sizes ≥0.50 as medium effects, and effect sizes ≥0.80 as large effects.

## Results

### Participant characteristics

Of the 418 students enrolled in the required course across the two cohorts, a total of N = 325 chose to participate in the present research evaluation (78%), suggesting favorable generalizability. Specifically, initial research enrollment rates were 88% in Cohort 1 (186/211 eligible students enrolled) and 67% in Cohort 2 (139/207 enrolled). Retention rates at 6-month follow-up were 65% in Cohort 1 (120/186) and 78% in Cohort 2 (109/139). Frequencies, means, and standard deviations for select demographic variables are presented in Table 1 and for all outcome measures in Supplementary Material. To assess for differences in student demographics across the two cohorts, we completed independent samples t-tests for continuous variables and chi-square tests for categorical variables. There were significant differences in endorsement of current mental illness (*χ*^2^(2) = 10.69, *p* = .005) and personality traits of agreeableness (*t*(293) = 2.59, *p* = .01) and emotional stability (*t*(290) = 2.94, *p* = .004) between cohorts. We controlled for these differences by inclusion of these variables in the final linear mixed models. All other baseline individual characteristics were consistent across cohorts.

**Table 1.** Descriptive statistics for demographics and covariates.

	Cohort 1 standard (N = 186)	Cohort 2 adapted (N = 139)	
	N (%)	N (%)	*χ*^2^(df), *p*
**Gender identity**			5.78(3), 0.123
Woman	79 (43)	70 (50)	
Man	104 (56)	66 (47)	
Other/prefer not to answer	3 (2)	2 (1)	
**Race/Ethnicity**			3.05(6), 0.802
Asian/Asian-American	37 (20)	27 (19)	
Hispanic/Latino	2 (1)	4 (3)	
Middle Eastern/North African	4 (2)	1 (0.72)	
Multiracial	6 (3)	1 (0.72)	
White/European-American	132 (71)	105 (76)	
Other/prefer not to answer	4 (2)	5 (4)	
**Current mental illness identification**			10.69(2), 0.005
Yes	38 (20)	46 (33)	
No	134 (72)	88 (63)	
Prefer not to answer	13 (7)	4 (3)	
	Mean (SD)	Mean (SD)	*t* (df), *p*
**Age**	27.14 (2.57)	26.93 (2.18)	0.79 (313), 0.432
**Personality traits**			
Agreeableness	5.25 (0.98)	4.96 (1.00)	2.59(293), 0.010
Emotional stability	5.04 (1.12)	4.67 (1.15)	2.94(290), 0.004

Additional response options were available as follows for self-identified gender identity and race/ethnicity, yet were not endorsed by any participants. Gender identity: Transgender Man, Transgender Woman; Race/ethnicity: American Indian/Alaska Native, Black/African American, Native Hawaiian or other Pacific Islander.

### Primary outcomes

**Figure 1.**
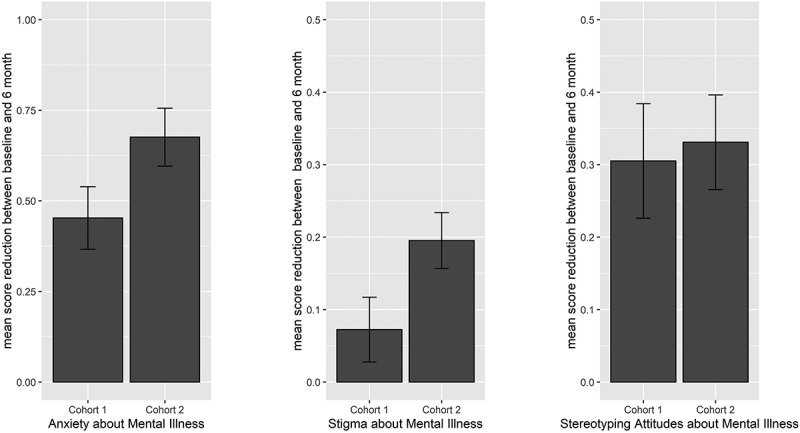
Figure depicts improvement through decreases in average scores. Bars represent average reduction from baseline to 6-months on the following outcomes, comparing Cohort 1 to Cohort 2: a) anxiety regarding interacting with an individual with mental illness (DMISS), b) stigma about mental illness (OMS), and c) stereotyping attitudes about mental illness (Characteristic Scale). Error bars display the standard errors of the mean difference. All changes are significant at an adjusted *p*-value cutoff of <0.05, except for Cohort 1 ‘Stigma about Mental Illness.’.

**Figure 2.**
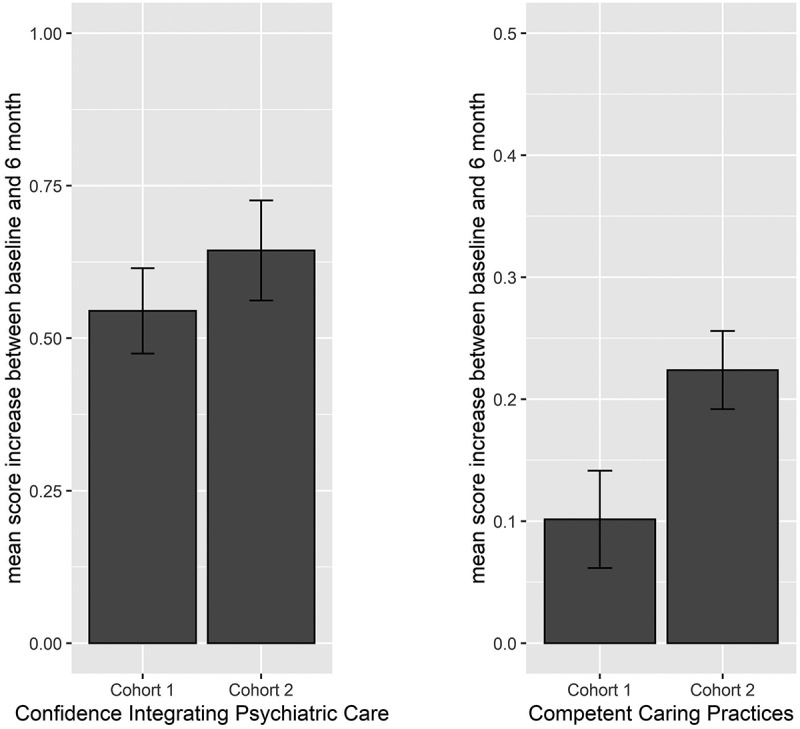
Figure depicts improvement through increases in average scores. Bars represent average increase from baseline to 6-months on the following outcomes, comparing Cohort 1 to Cohort 2: a) confidence integrating psychiatric care (ACIP) and b) competent caring practices (CCQ). Error bars display the standard errors of the mean difference. All changes are significant at an adjusted *p*-value cutoff of <0.05.

Across cohorts, longitudinal improvements in each of the three target domains of affect, beliefs, and behavioral intentions toward individuals with MI were consistently identified at the 6-month follow-up. In addition, effect sizes with the adapted curriculum were larger and observed across all six outcome measures (see detailed results in Table 2 and Figures 1 and 2). Of note, improvements were represented by either reductions in unwanted outcomes or by increases in desired outcomes. In the domain of *affect*, students reported reduced anxiety concerning interacting with someone with a mental illness. In the domain of *beliefs*, students demonstrated reductions in stereotyping negative attitudes towards patients with MI and, in Cohort 2 only, reductions in stigma. In the domain of *behavioral intentions*, students demonstrated increased confidence integrating psychiatric practice into routine medical care and improvements on a vignette-based measure of behavior demonstrating competent caring for mental health in a general medical setting. Furthermore, in Cohort 2 there were improved behavioral intentions regarding managing mental health during routine medical care that were not observed in Cohort 1.

**Table 2.** Improvements in affect, beliefs, and behavioral intentions regarding mental illness, from baseline to 6-month follow-up.

Domain measure	Cohort 1 standard (N = 186)	Cohort 2 adapted (N = 139)
**Affect**		
Anxiety about interacting with MI	−0.45	−0.68
	*p* < 0.0001	*p* < 0.0001
	ES = 0.49	ES = 0.80
**Beliefs**		
Stigma toward patients with MI	−0.07	−0.20
	*p* = 0.19	*p* < 0.0001
		ES = 0.45
Stereotyping attitudes about MI	−0.31	−0.33
	*p* = 0.0002	*p* < 0.0001
	ES = 0.38	ES = 0.46
**Behavioral intentions**		
Confidence in ability to integrate psychiatry in routine medical care	+0.54 *p* < 0.00001	+0.64 *p* < 0.0001
	ES = 0.66	ES = 0.92
Competent caring for MI in medical setting	+0.1 *p* = 0.0195	+0.22 *p* < 0.0001
	ES = 0.27	ES = 0.58
Intentions to manage MI in routine care	−0.00 *p* = 0.78	−0.05 *p* = 0.0049 ES = 0.32

All changes are significant at an adjusted p-value cutoff of <0.05, except for Cohort 1 ‘Stigma toward patients with MI’ and ‘Intentions to manage MI in routine care.’ All effects represent improvement, with (-) denoting decreases in unwanted outcomes and (+) denoting increases in desired outcomes. Analyses controlled for gender identity, race/ethnicity, socioeconomic status, current endorsement of having a mental illness, history of seeking mental health treatment, medical specialty interest in psychiatry, medical specialty interest in family medicine/primary care, modality of psychiatry clerkship, and personality traits.

MI = mental illness. ES = effect size: low ≥ 0.20; medium ≥ 0.50 and large ≥ 0.80.

In sum, the NAMI PEP consistently demonstrated longitudinal effectiveness when delivered as a curricular requirement across two cohorts of third-year medical students, with enhanced effects on affect, beliefs, and behavioral intentions regarding MI after adapting the program for medical students (Cohort 2). The adapted NAMI PEP had the largest impact on the domains of behavioral intentions and affect regarding MI, with large Cohort 2 effect sizes observed for increased confidence integrating psychiatry into routine care and reduced anxiety interacting with someone with MI (see Table 2). The largest effect size increases from Cohort 1 to Cohort 2 were on scales measuring principles of competent caring for MI and anxiety about interacting with someone with MI (see Table 2).

### Discussion

This study evaluated longitudinal outcomes of the NAMI PEP when implemented as a curricular requirement across two cohorts of third-year medical students, comparing standard implementation of the existing community program (Cohort 1) with an adapted program tailored to medical students (Cohort 2). The NAMI PEP resulted in either reductions in unwanted affective and attitudinal outcomes, or improvement in desired behavioral outcomes, with enhanced impact following its adaptation for medical students (Cohort 2). Six months after the NAMI PEP, students were less anxious about interacting with patients with mental illness, had less stigma and stereotyping negative attitudes about mental illness, reported more confidence integrating psychiatric practice into routine medical care, and demonstrated increased competence in principles of collaborative mental health treatment in a general practice setting. As longitudinal results were derived from two separate cohorts with their own respective baseline reference points, the consistent findings of direct improvements in target outcomes increase confidence in the effectiveness of the intervention and provide support for the adaptations made for medical students.

This study expands existing knowledge of contact-based psychiatry education in meaningful ways. First, it demonstrates the pragmatic longitudinal effectiveness of the NAMI PEP when integrated as a medical school curricular requirement. In showing that the NAMI PEP offers benefits for an array of students, not only those who voluntarily seek it as an elective educational opportunity, the results suggest potential broad workforce impact for future physicians across a range of medical specialties. This has important implications for provision of quality medical care for individuals with comorbid psychiatric illness. Further, the study demonstrates the feasibility of adapting an existing community-based program for contemporary medical education, with updates including a flipped classroom model, enhanced student participation and small group discussion, integration with other educational experiences, physician modeling, and virtual delivery. Together, these findings demonstrate the external validity and generalizability of the results under realistic medical education conditions.

By directly comparing the same contact-based psychiatry education program implemented in-person versus virtually using the same multidimensional outcome measures, this study also extends existing evidence that virtual interactions are a meaningful means of contact-based psychiatry education. Live virtual contact in lieu of in-person contact increases dissemination potential of such programs and may be more feasible for institutions with a distributed clerkship model, students who are geographically isolated from institutions during clerkships, or implementation with specific stigmatized patient populations. Additionally, videoconferencing features may increase the diversity of interaction opportunities to better engage certain learners (e.g., chat, polling, and whiteboard features) and provide some with enhanced psychological safety (e.g., anonymous submission of questions or feedback; virtual breakout room discussions) [14,21].

Further, the demonstration of magnified and sustained improvements 6 months after the program suggests longer-term benefits of the NAMI PEP than previously identified [22]. This highlights the curriculum’s potential to promote lasting changes in perspectives on and management of psychiatric illness, strengthening the promise for extension of benefits into students’ future practice as physicians. Demonstration of large and sustained improvements in behavioral intentions is especially critical, given the role of behavioral intentions in predicting future behavior and the limited assessment of behavioral outcomes in past studies. The adapted program (in Cohort 2) had particular impact in this domain, suggesting that the increased case examples, application to clerkship experiences, and contact with more senior physician role models may especially influence students’ ability to integrate principles of collaborative psychiatric care into practice.

The present study is not without limitations. We primarily relied on student self-report, which may be subject to demand characteristics. Although a practical challenge throughout contact-based education research [16], assessments of changes in student behavior (e.g., objective behavioral ratings of patient interactions; patient satisfaction ratings) would improve the study conclusions. Additionally, although our 6-month follow-up is a notable extension of the evaluation period in most of the literature on contact-based education [16], future research could extend the follow-up evaluation period further.

Our multiyear comparison across two cohorts is also limited by differential research enrollment, retention, and participant characteristics. Cohort 2 had a lower rate of research enrollment, yet improved participant retention as compared to Cohort 1. Ultimately, this resulted in comparable sample sizes at the primary 6-month follow-up across both cohorts. The use of a pre-recorded recruitment message in Cohort 2 (due to changes necessitated by the flipped classroom approach) may have impacted enrollment. Despite a strong overall enrollment rate across the two cohorts, it is possible that students who enrolled in the research differed from those who did not. Further, more students endorsed lived experience of mental illness in Cohort 2 than Cohort 1. Given past research suggesting that lived experience of mental illness is associated with reduced mental illness stigma [30], our analyses controlled for this difference (and other theoretically related differences) across cohorts. However, it is possible that there were other unmeasured differences between participants in the two cohorts that may have contributed to changes over time.

Future research should examine the generalizability of findings to other medical institutions. Although this represents the first known integration of the NAMI PEP into a medical school curriculum, the standardized nature of the curriculum and existing national infrastructure of local NAMI affiliates lends itself naturally to extension to other institutions in the USA. Still, the 15-hour length of the course may limit its implementation at many institutions. Although we chose to hold the course in condensed form across 2 days, there is flexibility to the delivery model. For example, the NAMI PEP can be subdivided into multiple smaller sessions, such as for offering multiple 2–3-hour sessions throughout a psychiatry clerkship. Moreover, future directions of our research program include empirical evaluation of a condensed, 4-hour NAMI PEP seminar offering an abbreviated version of content from this 15-hour program. The condensed nature of this program may increase its implementation potential.

Examination of ways to further streamline the NAMI PEP implementation to optimize dissemination and scalability in medical schools, without detracting from its benefits, is ongoing. Future directions also include expanding the program and its evaluation beyond medical students. The NAMI PEP educational benefits may generalize to other health professional students, increasing the potential scope of contact-based psychiatry education to a broader cross-section of the future health-care workforce while promoting collaborative interprofessional care of patients with mental illness.

Individuals living with mental illness face barriers to receiving competent medical care, contributing to health disparities such as increased morbidity and mortality. In addition to systemic issues such as limited access to specialty psychiatry services or integrated primary and behavioral health care, there is a critical need to increase all medical providers’ competencies and reduce stigma when working with this patient population [4]. These findings suggest that embedding a structured, contact-based intervention at the end of the third year of medical school is a highly effective means of improving affect, beliefs, and behaviors relevant to caring for patients with mental illness up to at least 6 months after the program. This longitudinal impact is promising for facilitating increasingly skilled provision-of-care in future interactions with patients with psychiatric illnesses.

## Data Deposition

The data that support the findings of this study are openly available in Harvard Dataverse at https://doi.org/10.7910/DVN/S0OKV5.
